# Impact of water exhaled out by visitors in show caves: a case study from the Moravian Karst (Czech Republic)

**DOI:** 10.1007/s11356-024-32946-2

**Published:** 2024-03-19

**Authors:** Marek Lang, Jiří Faimon, Pavel Pracný, Jindřich Štelcl, Sandra Kejíková, Jiří Hebelka

**Affiliations:** 1 Department of Geological Sciences, Faculty of Science, Masaryk University, Kotlářská 2, 611 37 Brno, Czech Republic; 2 Czech Geological Survey, Brno Branch, Leitnerova 22, 658 69 Brno, Czech Republic; 3 Department of Biology, Faculty of Education, Masaryk University, Poříčí 623/7, 603 00 Brno, Czech Republic; 4 Cave Administration of the Czech Republic, Svitavská 11, 678 01 Blansko, Czech Republic

**Keywords:** Exhaled water vapor/CO_2_, Show cave, Visitors, Speleothems, Calcite dissolution

## Abstract

The anthropogenic impact of the water and CO_2_ exhaled by visitors was studied in the show caves of the Moravian Karst (Czech Republic), especially in the Balcarka and Výpustek Caves. Two alternative models based on (1) the known/presumed composition of the breathed air and physical activity of visitors and (2) the detailed monitoring microclimatic data were proposed. The CO_2_ fluxes of 2.4 × 10^−4^ and (2.0–3.9) × 10^−4^ mol person^−1^ s^−1^ and the water vapor fluxes of (3.2–8.9) × 10^−3^ and (0.6–1.2) × 10^−2^ g person^−1^ s^−1^ were found for a slightly increased physical load. The total attendance and cave tour duration were the main driving factors. For the available data on attendance and accessibility periods, the total mass of water vapor exhaled by visitors in all show caves in the Moravian Karst was estimated between 9.6 × 10^6^ and 4.3 × 10^8^ g with significant seasonality. According to the geochemical model, this mass of water is capable of dissolving 1280 to 59,038 g of calcite, assuming a mean winter and summer CO_2_ concentration in the cave air of 1000 and 3000 ppmv. The larger extent of water condensation can lead to the so-called condensation corrosion, whereas the lower extent of condensation probably causes a recrystallization of calcite on the surface of speleothems and rocks.

## Introduction

The carbonate karst landscapes cover about 10–15% of the Earth’s land surface (Ford and Williams 2007; Panno et al. 2019) and represent an essential atmospheric CO_2_ sink due to carbonate rock weathering (Gaillardet et al. 2019). However, the observed disequilibrium between surface conditions and carbonate rocks indicates that karst areas are unstable systems and are denudated by water infiltrating into the karst profile during/after precipitation events (Dreybrodt 1988; Stumm and Morgan 1996). The development of a complete surface and subsurface karst system requires a relatively short geological period of hundreds of thousands of years (White 1988; Korpas 1998; Granger et al. 2001). Its gradual extinction is associated with irreversible dissolution by percolating karst water. During the last decades, anthropogenic activities have increasingly influenced the karst areas in multiple ways (Liu et al. 2018; Ravbar et al. 2021; Wang et al. 2022). Protection of karst areas such as water reservoirs/biotopes, cultural heritage, destination of tourism, and scientific information is very important. Minimalization of anthropogenic impacts is a lively topic of discussion among conservationists and environmentalists. Show caves are often discussed as the most affected sites. Being among the most highly admired and visited destinations in karst areas causes significant disturbances to the cave environment (see Lang et al. 2017b for a short review of these issues). One of the most discussed questions in this context is speleothem destruction through condensation corrosion (Sarbu and Lascu 1997; Dublyansky and Dublyansky 1998; Tarhule-Lips and Ford 1998; de Freitas and Schmekal 2006). In this case, exhaled water vapor, much warmer than solid surfaces (Mansour et al. 2020), condenses on the cave walls and speleothems. Such water is undersaturated with respect to calcite and corrodes calcite. Such an effect is clearly understudied. Therefore, some attempts have been made to propose more environmentally friendly cave management (see, e.g., Lobo 2015 or Šebela et al. 2019). Currently, the attention of cave researchers is primarily focused on the impact of anthropogenic CO_2_ on the cave environment (Trinh et al. 2018; Guirado et al. 2019; Surić et al. 2021). Obvious outstanding questions include (1) the extent of condensation of anthropogenic water vapor in the cave environment and (2) the combined effect of condensed anthropogenic water and exhaled anthropogenic CO_2_ on cave speleothems. This study is based on (i) new data sets from the Balcarka Cave and the Výpustek Cave (Moravian Karst) and (ii) dynamic microclimate and geochemical models. The purpose of this study was (1) to analyze the extent of breathed air, (2) to estimate water condensation, (3) to estimate the effect of the anthropogenic water on limestone tablets, and (4) to quantify this effect for all the show caves in Moravian Karst.

## Site of study

The Moravian Karst (MK) is the largest (total area of 94 km^2^) and the most extensive karst area in the Bohemian Massif (Czech Republic) and represents a part of the Drahany Highlands. The crystalline basement is formed by the Proterozoic granitic rocks of the Brno Crystalline Massif, covered by Devonian sandstone and conglomerate. During the Eifelian to Frasnian stages, these basal clastic sediments were overlain by a complex of more than 1000-m-thick limestones of the Macocha Formation consisting of the Vavřinec Limestone, Josefov Limestone, Lažánky Limestone, and Vilémovice Limestone. The overburden of the Macocha Formation represents several tens of meters thick Líšeň Formation evolved during the Famennian to the Middle Viséan stages. It is created by Křtiny Limestone and Hády-Říčka Limestone. The altitude of the karst plateau varies between 244 and 613 m above sea level (a.s.l.) with an average value of 447.5 m a.s.l. Based on the climate classification (Kottek et al. 2006), the MK is a moderately warm (average annual temperature 8.3 °C) and moderately humid (average annual rainfall 543 mm) climatic region with drier and warmer sites in its southern part. More than 1600 caves have been documented in the karst area, and five of them have been open to the public: the Balcarka Cave (BC), the Kateřinská Cave (KC), the Punkva Caves (PC), the Sloup-Šošůvka Caves (S-ŠC), and the Výpustek Cave (VC). A summary of the parameters of individual show caves is presented in Table 1.

**Table 1:** Parameters of show caves. The visitor tour information is valid as of 2021

Cave	Length (m)		Tour duration (min)	Seasonal attendance	Acronym
	Cave totally	Visitor route			
Balcarka Cave	1150	720	60	23,636 to 57,435	BC
Kateřinská Cave	950	580	40	33,183 to 85,107	KC
Punkva Cave	6375	1250	60	121,738 to 293,377	PC
Sloup-Šošůvka Cave	5700	1760	110	30,867 to 59,099	S-ŠC
Výpustek Cave	2000	600	75	16,968 to 29,276	VC

BC and VC were chosen as monitoring sites for primary research due to different attendance during the late fall. While the BC visitor regime did not exceed 100 people/day, almost 1000 people/day passed through the VC during this period. Due to the similar climatic conditions and CO_2_ concentrations during the whole season (Lang et al. 2015b, 2017b, c), the observed phenomenon was subsequently modeled for conditions in all show caves in MK based on the new data sets.

**Fig. 1:**
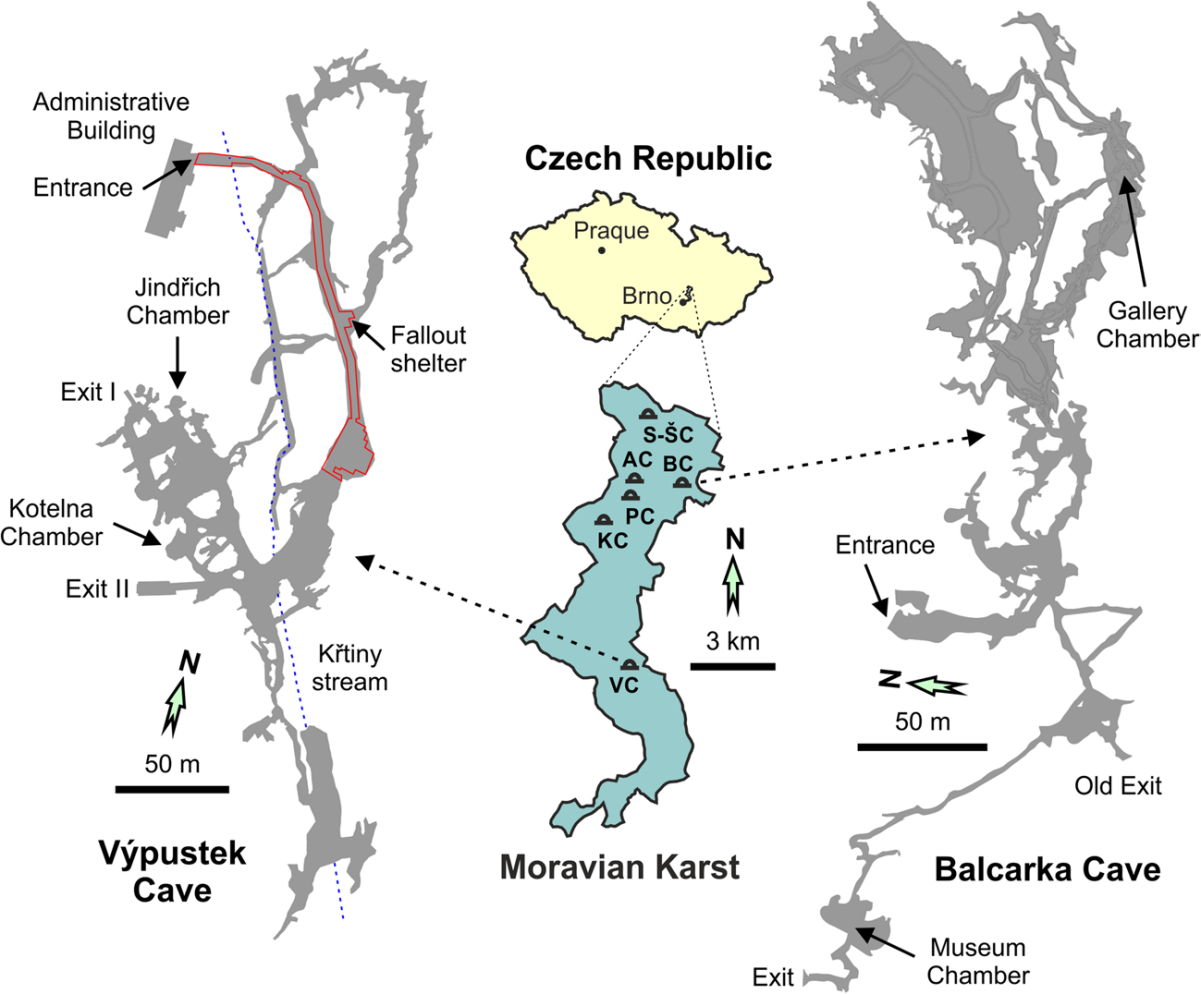
The position of individual show caves in the Moravian Karst and sketch maps of the monitoring sites. The acronyms correspond to Sloup-Šošůvka Caves (S-ŠC), Amatérská Cave (AC), Balcarka Cave (BC), Kateřinská Cave (KC), Punkva Caves (PC), and Výpustek Cave (VC). See text for details

The BC is situated in the northern part of the MK near the village of Ostrov u Macochy and consists of two levels of narrow corridors (total length of 1150 m) and chambers with rich speleothem decoration. The height distance between both cave levels is ~ 20 m, and the thickness of the overburden reaches 40 m. The same distance is probably between the levels of the lowest opening (444.3 m) and the mouth of the Discover’s Chimney (483.2 m) representing the highest opening. The cave shows a complex morphology characterized by alternation of ascending/descending passages connected with the exterior by three known entrances at different altitudes of 459.3 m (main entrance), 457.5 m (old exit), and 446.9 m (main exit) (Lang et al. 2015b). Such morphology ensures a typical dynamic behavior of air circulation. The total attendance in BC varies between 20,000 and 60,000 people/year. The Museum Chamber (458.4 m) situated near the old exit was chosen as the monitoring site (Fig. 1).

VC is located in the central part of the MK, about 2 km from the village of Křtiny. The cave represents a complex of relatively narrow corridors and large chambers formed by the Křtiny Stream, totaling 2000 m. It consists of two levels and three entrances at an altitude of 385 m, and the thickness of the overburden reaches 55 m. A complex morphology of the cave ensures typical dynamic air circulation. Between 1961 and 2001, an underground fallout shelter and a secret command post operated in the cave. Since 2008, the cave has been open to the public, with a total attendance of 16,000 to 30,000 people annually. The Jindřich Chamber and the Kotelna Chamber in deeper cave passages were chosen as the monitoring sites (Fig. 1).

## Methods

### Schedule of the monitoring campaigns

Data were collected during various monitoring campaigns conducted under different visitor regimes between November 2019 and October 2022. Results of the four campaigns carried out under a low-number visitor regime and a large-number visitor regime are presented. The schedule of these campaigns is given in Table 2.

**Table 2:** Monitoring schedule

Campaign	Date	Site	Period (hours)	Number of tours	Visitors totally
Abbreviation					
Balcarka Cave					
BC-C1	22-Oct-22	Museum Chamber	10.0	7	89
BC-C2	20-Nov-19	Museum Chamber	2.5	4	32
Výpustek Cave					
VC-C1	30-Nov-19	Kotelna Chamber	10.0	8	794
VC-C2	30-Nov-19	Jindřich Chamber	10.0	8	794

The campaigns in the Balcarka Cave were carried out under the low-number visitor regime. Whereas BC-C2 represented a reference campaign in the Museum Chamber controlled by strictly defined conditions providing a standard against which other measurements can be compared, the campaign BC-C1 continued in the Museum Chamber during a standard visiting regime. Unlike the measurements in Balcarka Cave, the campaigns in Výpustek Cave occurred in the Kotelna Chamber (VC-C1) and Jindřich Chamber (VC-C2) with an extreme number of visitors.

The visitor regime conditions for the BC-C2 reference campaign were defined to quantify the anthropogenic impact on the chamber environment. The controlled regime consisted of the entry of four eight-person tours at defined intervals and their stay in the chamber for specified periods. During other campaigns, the conditions were not constrained. The number of visitors in individual tours was recorded before entering the cave. The entries into the monitoring chamber were controlled by the relative speed of individual guided tours separated by 57 to 70 min (campaign BC-C1), 8 to 17 min (campaign VC-C1), and 29 to 30 min (campaign VC-C2). The visitors then stayed in the chamber for 2–7 min (campaign BC-C1), 9–21 min (campaign VC-C1), and 30 min (campaign VC-C2). The initial interaction time of the visitors with the chamber environment and the duration of stay during all monitoring campaigns were fine-tuned to be consistent with the temperature peak.

### Monitored variables

In addition to monitoring visitor traffic, BC-C1, BC-C2, and VC-C1 campaigns included monitoring CO_2_ concentrations, temperature, and relative humidity with minute time steps in both the cave air and the external atmosphere. In the case of the VC-C2 campaign, only the temperature and relative humidity values were recorded. All variables were measured at 2 m height above the surface/floor. The measurement setup consisted of an external datalogger (ALMEMO 2290–4 V5, Ahlborn, Germany) and individual sensors (all the sensors were connected with a single external datalogger). The CO_2_ concentration was measured with a handheld device (FYAD00CO2B10 digital sensor) with a measuring range from 0 to 10,000 ppmv and accuracy ± 100 ppmv + 5% of the measured value. The sensor FHA646E1, Ahlborn, Germany (± 0.4 °C in the range from − 20 to 0 °C, ± 0.1 °C in the range from 0 to 70 °C), was used to determine the cave air temperature, *T*_cave_. Relative humidity, *RH*, was measured with the manual humidity sensor FNAD46, Ahlborn, Germany (measuring range from 10 to 100% and accuracy ± 1%), except for the campaign VC-C1, where the *RH* values were recorded by the same sensor as the air temperature. For modeling, the *RH* values in (%) were recalculated into absolute humidity values, *AH*, (g m^−3^) based on the Flatau polynomial (Flatau et al. 1992).

1 $${\text{AH}}={\text{RH}}\left\{\sum\nolimits_{i=0}^{i=6}{{\text{a}}}_{i}\left(T-{T}_{0}\right)\right\}/100,$$

where *AH* is the absolute humidity (g m^−3^), RH is the relative humidity (%), the term between brackets represents the partial pressure of saturated water vapor (Pa), *a*_*i*_ are the coefficients of Flatau’s polynomial, *T* is the temperature (K), and *T*_0_ = 273.15 K. External temperature, *T*_ext_, was recorded using the COMET S3120 datalogger (TR Instruments Inc., Czech Republic, measuring range from − 30 to 80 °C with precision of ± 0.4 °C).

### Data analysis

#### Dynamic model

For modeling the evolution of the cave CO_2_ concentration, the Museum Chamber was considered a perfectly mixed reactor represented by a homogeneous reservoir with input and output fluxes. The CO_2_ fluxes (in mol s^−1^) include (1) the advective input flux from the exterior or/and an adjacent cave space, ${j}_{{\text{in}}}$, (2) the anthropogenic flux, ${j}_{A}$ (resulting from human respiration), and (3) the advective output flux out of the chamber, ${j}_{{\text{out}}}$. Following the model of the evolution of CO_2_ concentration, the model of absolute humidity evolution was proposed. The absolute humidity model was advanced by the flux associated with the condensation of anthropogenic water vapor (derived from human breathing), ${j}_{C}$. Since absolute humidity represents the concentration of water vapor, the absolute humidity modeling was based on the balance of individual water vapor fluxes.

For the modeling of anthropogenic water vapor produced by visitors in the show caves, BC-C2 was used as a reference campaign. Subsequently, the resulting model parameters were applied to the BC-C2 data to verify the results. The modeling consisted of two steps: simulation of (i) the evolution of the CO_2_ levels and, consequently, (ii) the evolution of the absolute humidity. The model parameters obtained from the CO_2_ level simulation (total volume, volumetric airflow velocity) were subsequently used to simulate the evolution of absolute humidity.

The instantaneous CO_2_ concentration in the chamber atmosphere, ${j}_{{{\text{CO}}}_{2}}$, is given by the sum of all individual fluxes:

2 $${j}_{{{\text{CO}}}_{2}}\text{=}\frac{{\text{d}}{\text{n}}_{{{\text{CO}}}_{2}}}{\text{dt}}\text{=}\frac{{\text{Vd}}{{\text{c}}}_{{{\text{CO}}}_{2}}}{\text{dt}}\text{=}\sum\nolimits_{i}{j}_{i},$$

where ${n}_{{{\text{CO}}}_{2}}$ is the total content of CO_2_ in the chamber atmosphere (mol), *t* is time (s), *V* is the chamber volume (m^3^), ${c}_{{{\text{CO}}}_{2}}$ is the instantaenous CO_2_ concentration in the chamber atmosphere (mol m^−3^), and *j*_*i*_ are CO_2_ fluxes (mol s^−1^).

The advective fluxes ${j}_{{\text{in}}}$ and ${j}_{{\text{out}}}$, respectively, were expressed as a product of CO_2_ concentration and airflows:

3 $${j}_{\mathrm{in }}= \text{ } {\text{v}} \, {c}_{{{\text{CO}}}_{2}}^{{\text{adj}}}$$

and

4 $${j}_{{\text{out}}}=v {c}_{{{\text{CO}}}_{2}}$$

where *v* is the volumetric velocity of the airflow through the chamber (m^3^ s^−1^) and *c*_*i*_ represents CO_2_ concentration in the atmosphere of an adjacent chamber (${c}_{{{\text{CO}}}_{2}}^{{\text{adj}}}$) and Museum Chamber (${c}_{{{\text{CO}}}_{2}}$), both in (mol m^−3^).

The anthropogenic flux, ${j}_{A}$, was defined as

5 $${j}_{A }= \text{ } {\text{A}} \, {j}_{{{\text{CO}}}_{2}}^{{\text{AP}}2},$$

where *A* is the attendance (number of visitors) and ${j}_{{{\text{CO}}}_{2}}^{{\text{AP}}2}$ is personal CO_2_ flux (mol person^−1^ s^−1^).

Inserting all CO_2_ fluxes into Eq. (2) gives

6 $$\frac{{{\text{dc}}}_{{{\text{CO}}}_{2}}}{{\text{dt}}}=\frac{v {c}_{{\text{CO}}_2}^{{\text{adj}}}}{V}+\frac{A {j}_{{{\text{CO}}}_{2}}^{{\text{AP}}2}}{V}-\frac{v {c}_{{\text{CO}}_2}}{V}.$$

Equation (6) was integrated under the condition that the parameters *v*, ${c}_{{{\text{CO}}}_{2}}$, *A*, and ${j}_{{{\text{CO}}}_{2}}^{{\text{AP}}2}$ are constants. The resulting equation for the cave CO_2_ concentrations is

7 $${c}_{{{\text{CO}}}_{2}}={c}_{0}{ e}^{-\frac{v}{V} t}+{c}_{{{\text{CO}}}_{2}}^{{\text{adj}}}\left(1-{e}^{-\frac{v}{V} t}\right)+\frac{A}{v} {j}_{{{\text{CO}}}_{2}}^{{\text{AP}}2} \left(1-{e}^{-\frac{v}{V} t}\right).$$

The maximum reachable concentration of CO_2_ under given conditions is consistent with the steady state. Such state is characterized by a balance of all the CO_2_ fluxes into/out of the chamber, which leads to the constant CO_2_ concentration in the chamber (${{\text{dc}}}_{{{\text{CO}}}_{2}}$/dt = 0). From Eq. (7), the natural steady-state CO_2_ concentration, ${c}_{{{\text{CO}}}_{2}}^{{\text{ss}}}$, results in

8 $${c}_{{{\text{CO}}}_{2}}^{{\text{ss}}}={c}_{{{\text{CO}}}_{2}}^{{\text{adj}}}+\frac{A {j}_{{{\text{CO}}}_{2}}^{{\text{AP}}2}}{v},$$

where all symbols have their standard meaning.

The instantaneous concentration of water vapor in the chamber atmosphere, ${j}_{{\text{WV}}}$, is given by the sum of all individual fluxes into/out of the chamber:

9 $${j}_{{\text{WV}}}\text{=}\frac{{\text{d}}{\text{n}}_{\text{WV}}}{\text{dt}}\text{=}\frac{{\text{Vd}}{{\text{c}}}_{{\text{WV}}}}{\text{dt}}\text{=}{ j}_{{\text{in}}}+ \text{ } {j}_{A}-{j}_{{\text{out}}}-{j}_{C},$$

where ${n}_{{\text{WV}}}$ is the total content of water vapor in the chamber atmosphere (g), *t* is time (s), *V* is the chamber volume (m^3^), and *j*_*i*_ are the water vapor fluxes (g s^−1^).

The fluxes ${j}_{{\text{in}}}$ and ${j}_{{\text{out}}}$ linked to the cave ventilation are expressed as

10 $${j}_{{\text{in}}}= \text{ } {\text{v}} \, {c}_{{\text{WV}}}^{{\text{adj}}}$$

and

11 $${j}_{{\text{out}}}=v {c}_{{\text{WV}}},$$

where *v* is the volumetric velocity of the airflow through the chamber (m^3^ s^−1^) and *c*_*i*_ corresponds to water vapor concentration in the atmosphere of an adjacent chamber (${c}_{{\text{WV}}}^{{\text{adj}}}$) and Museum Chamber (${c}_{{\text{WV}}}$), both in (g m^−3^).

The anthropogenic flux, ${j}_{A}$, corresponded to

12 $${j}_{A}= \text{ } {\text{A}} \, {j}_{{\text{WV}}}^{{\text{AP}}2},$$

where *A* is the attendance (number of visitors) and ${j}_{{\text{WV}}}^{{\text{AP}}2}$ represents personal water vapor flux (g person^−1^ s^−1^).

After integration of Eq. (9), the total increment in chamber water vapor is

13 $${c}_{{\text{WV}}}={c}_{0 }{e}^{-\frac{v}{V }t}+{c}_{{\text{WV}}}^{{\text{adj}}} \left(1-{e}^{-\frac{v}{V} t}\right)+\frac{A {j}_{{\text{WV}}}^{{\text{AP}}2 }- {j}_{C}}{v}\left(1-{e}^{-\frac{v}{V} t}\right).$$

For the steady state, water vapor concentration, Eq. (15) yields

14 $${c}_{{\text{WV}}}^{{\text{ss}}}={c}_{{\text{WV}}}^{{\text{adj}}}+\frac{A {j}_{{\text{WV}}}^{{\text{AP}}2}}{v}-\frac{{j}_{C}}{v},$$

where ${c}_{{\text{WV}}}^{{\text{ss}}}$ is the steady-state concentration of water vapor.

#### Statistical analysis

Obtained microclimatic data from monitoring (CO_2_ concentration, *T*, *RH*) were statistically processed in Statistica 14. The individual datasets were (i) recalculated to equidistant data and (ii) standardized via differencing using the equation *x* = *x* − *x*_(lag)_ with lag set to 1. The standardized equidistant data were subsequently analyzed using Spearman’s correlation.

#### Geochemical analysis

The potential amount of calcite, completely dissolved in condensed water in individual show caves of the Moravian Karst, was calculated based on the PHREEQC code with the default thermodynamic database (Parkhurst and Appelo 2013). It is important to note that the calculation was based on the assumptions that (1) all water vapor released by visitor exhalation completely condenses on the cave walls and (2) only primary calcite dissolution was considered (an effect of calcite recrystallization was out of the scope of this study). Parameters of the calculation were (i) the total volume of condensed water, (ii) ${P}_{{{\text{CO}}}_{2}}$ values in caves, and (iii) the theoretical amount of calcite dissolved in 1 L of water. Considering that not only anthropogenic CO_2_ participated in the dissolution of calcite, two typical values of ${P}_{{{\text{CO}}}_{2}}$, 10^−3.00^ and 10^−2.52^, for the winter and summer seasons, respectively, were chosen for the modeling based on the long-term range of cave values in the region (Lang et al. 2017c). The molar quantities were consequently recalculated to the mass.

#### Calculation of dew-point

The *T*_DP_ is commonly defined as the temperature of saturated air at actual water vapor concentration and is mainly affected by temperature and relative humidity. Many equations describing this relationship have been proposed. Lawrence (2005) found simple expression for moist air (*RH* > 50%), where the *T*_DP_ decreases by about 1 °C for every 5% decrease in *RH*:

15 $${T}_{{\text{DP}}}=T-\left(\frac{100-{\text{RH}}}{5}\right),$$

where *T* is the temperature (°C) and *RH* is the relative humidity (%).

#### Calculation of virtual temperature difference

The cave airflows are mostly related to the temperature difference between external and cave air temperature (Jernigan and Swift 2001; Kowalczk and Froelich 2010; Faimon and Lang 2013); however, it does not account for the composition of cave air. Therefore, the temperature difference could be replaced virtual temperature difference, $\Delta {T}^{{\text{virt}}.}$, calculated based on Sánchez-Cañete et al. (2013) as

16 $${\Delta {T}^{{\text{virt}}.}= T}_{{\text{ext}}}^{{\text{virt}}.}-{T}_{{\text{cave}}}^{{\text{virt}}.},$$

where ${T}_{{\text{ext}}}^{{\text{virt}}.}$ is virtual external temperature (°C) and ${T}_{{\text{cave}}}^{{\text{virt}}.}$ is the virtual cave temperature (°C). Note that the positive $\Delta {T}^{{\text{virt}}.}$ values correspond to the airflow from deeper cave passages into the monitored chamber (inflowing air) and the negative values to the direction from the monitored chamber to the external atmosphere. The positive values correspond to downward airflows (DAF ventilation mode), and the negative values correspond to upward airflows (UAF ventilation mode) (Faimon et al. 2012).

#### SEM analysis

Based on White et al. (2021), the water condensation effect was studied on the fresh limestone tablets of 6 × 4 × 0.5 cm in dimensions by SEM analysis. These tablets were cut from the Vilémovice-type limestone and placed into the Amatérská Cave (Moravian Karst) for a period of 30 months. The surface of the tablets was analyzed by a scanning electron microscope JEOL 6490LV equipped with an EDX microanalyzer (Oxford Instruments) in the Laboratory of Electron Microscopy and Microanalysis of the Department of Geological Sciences (Faculty of Science, Masaryk University in Brno). Before analysis, the sample surface was coated with gold. The limestone surface images were made in the mode of secondary electrons.

## Results

### Exhaled water by visitors in the showed cave of MK

Based on the number of visitors, the total volume of exhaled water was estimated for (1) the winter season (January to March, October to December), (2) the summer season (April to September), and (3) the whole season. The calculation was conducted for the accessibility periods and subsequently summed to express the total amounts of exhaled water. The calculations cover different total periods for individual caves, based on the availability of attendance data: 1955–2019 for PC; 1990–2019 for BC, KC, and S-ŠC; and 2007–2019 for VC (calculations were limited to 2019 due to the start of the COVID-19 pandemic). The duration of the individual cave tours used for the calculation was 40 min (PC), 60 min (BC, S-ŠC), 75 min (KC), and 110 min (VC). The volume of air exhaled was estimated for two physical activities: low activity with an exhaled frequency of 15 breaths min^−1^ and increased activity with a frequency of 30 breaths min^−1^. Mean volume of the breath was taken to be 0.5 L, and exhaled air was assumed to be saturated by water vapor at 37 °C. Since the cave wall is considered to be tempered on the temperature of cave air, after the chamber atmosphere reaches saturation with water vapor (*RH* = 100%), the supersaturated water vapor immediately condenses on the cooler cave wall. However, the transport of water vapor to the cave wall requires some time. An anthropogenic flux of breathed-out vapor, ${j}_{{\text{WV}}}^{{\text{AP}}1}$, corresponds to 5.8 × 10^−3^ g person^−1^ s^−1^ for the low activity and 1.2 × 10^−2^ g person^−1^ s^−1^ for the increased activity. The concentration of CO_2_ in exhaled air was estimated to be 40,000 ppmv, that is, 1.6 mol m^−3^. Based on previously given activities and mean breath volume, personal CO_2_ flux, ${j}_{{{\text{CO}}}_{2}}^{{\text{AP}}1}$, varied in the range of (2.0–3.9) × 10^−4^ mol person^−1^ s^−1^. Attendance during the total accessible period in individual show caves (12–64 years) ranged from 249,249 to 15,208,888 people, with a lower attendance of 58,124 to 1,841,708 people during the winter season and a higher attendance of 191,125 to 13,367,180 people during the summer season. In low visitors’ activity, the whole monitored periods were associated with exhaled air in the range of (0.2–4.6) × 10^6^ m^3^, leading to a release of 9.6 × 10^6^ to 2.1 × 10^8^ g of water vapor and condensation of 9.6 to 213.3 m^3^ of water. During the winter seasons, the exhaled air varying in the range of (0.3–5.5) × 10^5^ m^3^ caused the release of water vapor mass in the range of (0.2–2.6) × 10^7^ g and condensation of 1.5 to 25.8 m^3^ of water. However, higher attendance in the summer seasons reflected increased values of air volume (1.6 × 10^5^ to 4.0 × 10^6^ m^3^), water vapor mass (7.4 × 10^6^ to 1.9 × 10^8^ g), and volume of condensed water (7.4 to 187.5 m^3^). The results of the calculation for both regimes (15 and 30 breaths min^−1^) are given in Table 3.

**Table 3:** Total contents of exhaled air and water vapor in the single show caves of the Moravian Karst for available periods

Period	Season	Visitor number (person)	Low physical activity			Increased physical activity		
			Air vol. (m^3^)	WV mass (g)	W vol. (m^3^)	Air vol. (m^3^)	WV mass (g)	W vol. (m^3^)
Balcarka Cave (mean tour time = 60 min)								
1990–2019	Winter	69,566	3.1 × 10^4^	1.5 × 10^6^	1.5	6.3 × 10^4^	2.9 × 10^6^	2.9
1990–2019	Summer	996,021	4.5 × 10^5^	2.1 × 10^7^	21.0	9.0 × 10^5^	4.2 × 10^7^	41.9
1990–2019	Totally	1,065,587	4.8 × 10^5^	2.2 × 10^7^	22.4	9.6 × 10^5^	4.5 × 10^7^	44.8
Kateřinská Cave (mean tour time = 75 min)								
1990–2019	Winter	110,905	6.2 × 10^4^	2.9 × 10^6^	2.9	1.3 × 10^5^	5.8 × 10^6^	5.8
1990–2019	Summer	1,605,887	9.0 × 10^5^	4.2 × 10^7^	42.2	1.8 × 10^6^	8.4 × 10^7^	84.5
1990–2019	Totally	1,716,792	9.7 × 10^5^	4.5 × 10^7^	45.1	1.9 × 10^6^	9.0 × 10^7^	90.3
Punkva Caves (mean tour time = 40 min)								
1955–2019	Winter	1,841,708	5.5 × 10^5^	2.6 × 10^7^	25.8	1.1 × 10^6^	5.2 × 10^7^	51.7
1955–2019	Summer	13,367,180	4.0 × 10^6^	1.9 × 10^8^	187.5	8.0 × 10^6^	3.8 × 10^8^	374.9
1955–2019	Totally	15,208,888	4.6 × 10^6^	2.1 × 10^8^	213.3	9.1 × 10^6^	4.3 × 10^8^	426.6
Sloup-Šošůvka Caves (mean tour time = 60 min)								
1990–2019	Winter	103,826	4.7 × 10^4^	2.2 × 10^6^	2.2	9.3 × 10^4^	4.4 × 10^6^	4.4
1990–2019	Summer	1,162,534	5.2 × 10^5^	2.5 × 10^7^	24.5	1.1 × 10^6^	4.9 × 10^7^	48.9
1990–2019	Totally	1,266,360	5.7 × 10^5^	2.7 × 10^7^	26.6	1.1 × 10^6^	5.3 × 10^7^	53.3
Výpustek Cave (mean tour time = 110 min)								
2007–2019	Winter	58,124	4.8 × 10^4^	2.2 × 10^6^	2.2	9.6 × 10^4^	4.5 × 10^6^	4.5
2007–2019	Summer	191,125	1.6 × 10^5^	7.4 × 10^6^	7.4	3.2 × 10^5^	1.5 × 10^7^	14.7
2007–2019	Totally	249,249	2.1 × 10^5^	9.6 × 10^6^	9.6	4.1 × 10^5^	1.9 × 10^7^	19.2

*Air vol*. air volume, *WV mass* water vapor mass, *W vol.* water volume

### Calcite dissolution

In case of low visitors’ activity, the total amounts of dissolved calcite during the winter seasons varied in the range of 1.4 to 25.2 mol (143 to 2520 g); however, the values between 10.6 and 269.7 mol (1062 and 26,999 g) were registered in the summer seasons. In sum, it gives total amounts of dissolved calcite in the range of 12.8 to 294.9 mol (1280 to 29,519 g) for the whole accessibility period of individual caves. The values calculated for all mentioned conditions under the regime of increased visitors’ activity were doubled. The results of the calculation are given in Table 4.

**Table 4:** Hypothetical amounts of dissolved calcite by anthropogenic water in dependence on the attendance in the Moravian Karst show caves

Period	Season	Visitor number (person)	Low physical activity			Increased physical activity		
			W vol. (m^3^)	Dissolved calcite		W vol. (m^3^)	Dissolved calcite	
				(mol)	(g)		(mol)	(g)
Balcarka Cave								
1990–2019	Winter	69,566	1.5	1.4	142.8	2.9	2.9	286
1990–2019	Summer	996,021	21.0	30.1	3017.6	41.9	60.3	6035
1990–2019	Totally	1,065,587	22.4	31.6	3160.4	44.8	63.2	6321
Kateřinská Cave								
1990–2019	Winter	110,905	2.9	2.8	284.5	5.8	5.7	569
1990–2019	Summer	1,605,887	42.2	60.8	6081.7	84.5	121.5	12,163
1990–2019	Totally	1,716,792	45.1	63.6	6366.2	90.3	127.2	12,732
Punkva Caves								
1955–2019	Winter	1,841,708	25.8	25.2	2519.9	51.7	50.4	5040
1955–2019	Summer	13,367,180	187.5	269.7	26,998.9	374.9	539.5	53,998
1955–2019	Totally	15,208,888	213.3	294.9	29,518.8	426.6	589.8	59,038
Sloup-Šošůvka Caves								
1990–2019	Winter	103,826	2.2	2.1	213.1	4.4	4.3	426
1990–2019	Summer	1,162,534	24.5	35.2	3522.1	48.9	70.4	7044
1990–2019	Totally	1,266,360	26.6	37.3	3735.2	53.3	74.6	7470
Výpustek Cave								
2007–2019	Winter	58,124	2.2	2.2	218.7	4.5	4.4	437
2007–2019	Summer	191,125	7.4	10.6	1061.6	14.7	21.2	2123
2007–2019	Totally	249,249	9.6	12.8	1280.3	19.2	25.6	2561

Based on the PHREEQC calculations (Parkhurst and Appelo 2013). Winter value of CO_2_: 1000 ppmv; mass of dissolved calcite: 9.8 × 10^−4^ mol L^−1^. Summer value of CO_2_: 3000 ppmv; mass of dissolved calcite: 1.4 × 10^−3^ mol L^−1^

*W vol.* water volume

### Detailed monitoring in individual cave chambers

#### Monitoring under standard visiting regime

**Fig. 2:**
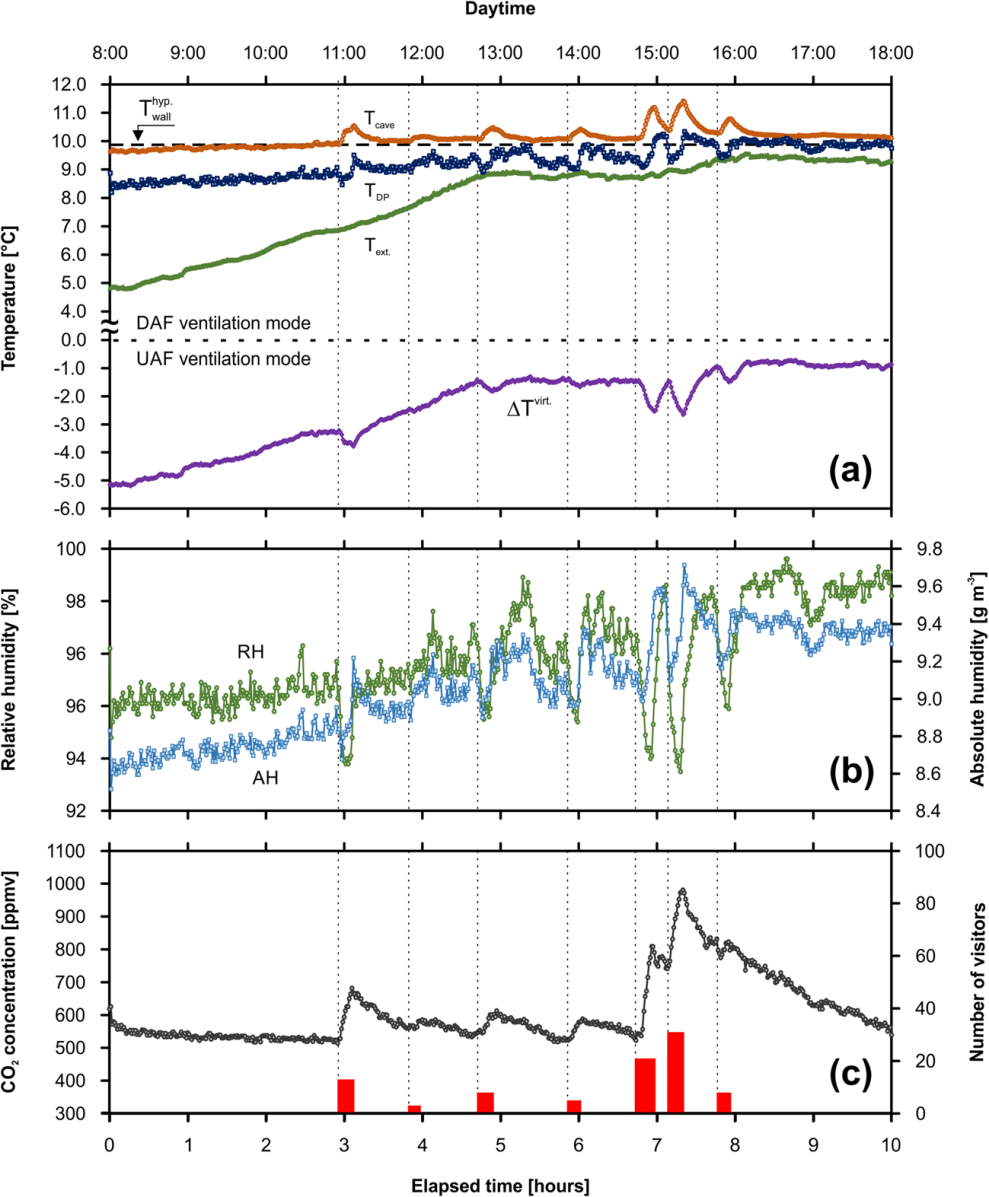
Monitoring campaign BC-C1 (22 October 2022; Museum Chamber, Balcarka Cave): the evolution of cave air/dew-point/external temperature and virtual temperature difference (**a**), cave air relative/absolute humidity (**b**), and CO_2_ concentration (**c**) in dependence on the individual tours of visitors (the widths of columns represent the time visitors spent in the chamber)

The monitoring campaign BC-C1 was carried out in the Museum Chamber (Balcarka Cave) under the standard visiting regime. It covered 89 people divided into 7 tours. Individual tours of visitors correlate positively with sharp peaks in the variables such as *T*_cave_, *T*_DP_, and CO_2_ concentrations, however negatively with *RH* (Fig. 2). Before starting the first cave visits, values of *T*_cave_, *T*_DP_, and *RH* gradually increased, whereas CO_2_ concentrations showed relatively constant values. The *T*_cave_ values increased from approximately 9.6 to 11.5 °C (individual increments ~ 0.2–1.1 °C) (Fig. 2a). Similar trend was observed in the case of *T*_DP_ values, where increments of 0.7–1.1 °C corresponding to individual visitor tours lead to the increase from the initial value of 8.2 °C to the peak value of 10.3 °C. Based on the *T*_ext_ values increasing from 4.8 to 9.5 °C, the $\Delta {T}^{{\text{virt}}.}$ values systematically increased from the initial value of − 5.2 to − 0.7 °C. The negative values of $\Delta {T}^{{\text{virt}}.}$ indicate the UAF ventilation mode during the whole campaign.

Since *RH* values are negatively dependent on air temperature, the anthropogenically increased cave air temperature caused decreases of up to 7.1% of the natural *RH* values varying between 91.5 and 99.6%. On the other hand, the visitors’ presence led to increases of more than 0.6 g m^−3^ for values of *AH* ranging from 8.5 to 9.7 g m^−3^ (Fig. 2b). Whereas the natural CO_2_ concentrations varied about relatively constant value of ~ 550 ppmv, the anthropogenically influenced CO_2_ concentrations reached up to 981 ppmv (Fig. 2c). Based on the number of visitors in individual tours, the net increases in CO_2_ ranged from 26 to 286 ppmv.

#### Monitoring under well-defined cave conditions

**Fig. 3:**
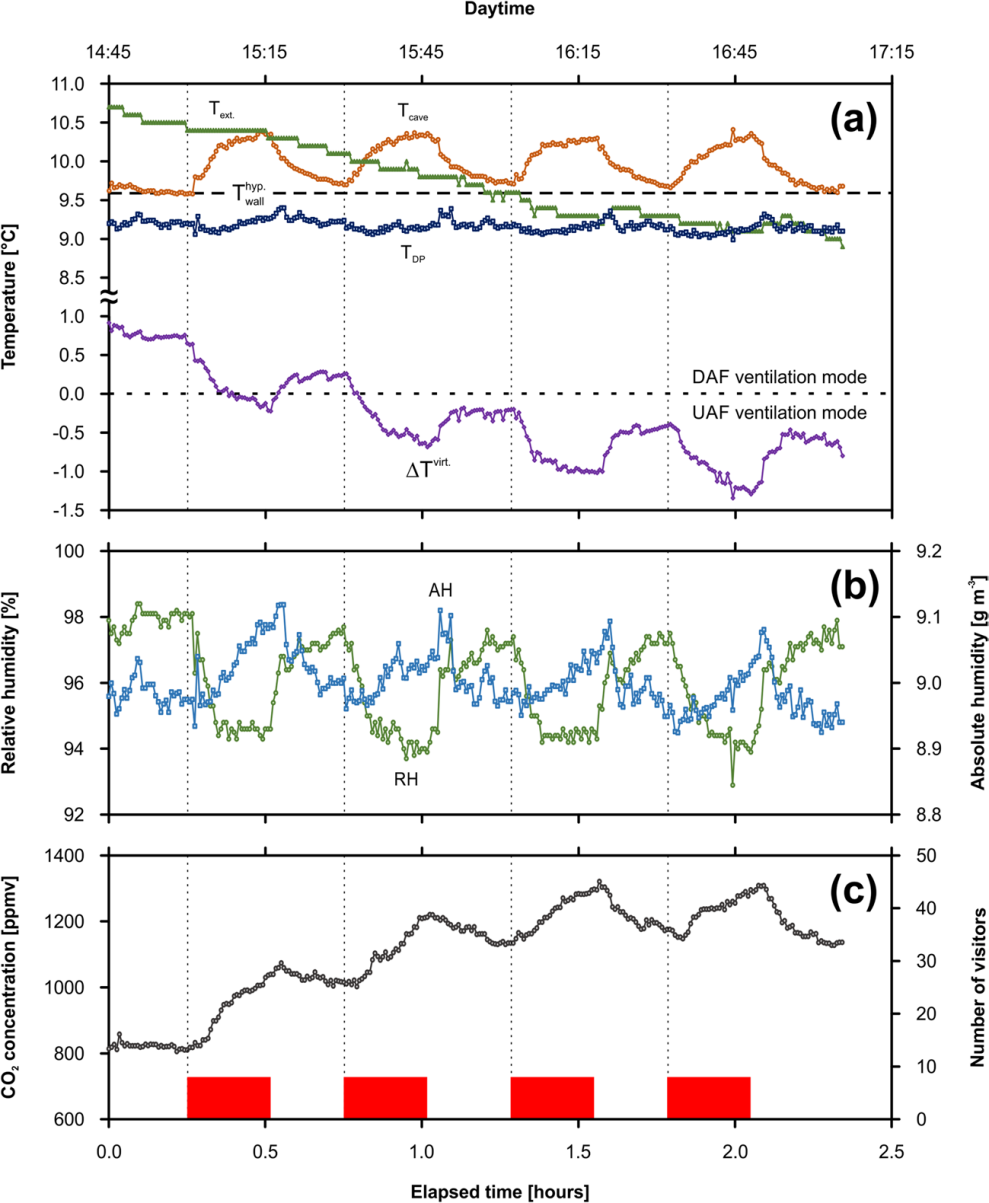
Reference monitoring campaign BC-C2 (20 November 2019; Museum Chamber, Balcarka Cave): the evolution of cave air/dew-point/external temperature and virtual temperature difference (**a**), cave air relative/absolute humidity (**b**), and CO_2_ concentration (**c**) in dependence on the individual tours of visitors (the widths of columns represent the time visitors spent in the chamber)

This monitoring was designed so that the results could be used to verify a simple dynamic model. For this purpose, the Museum Chamber (Balcarka Cave) with a well-defined volume that is bordered by two narrow corridors as chamber entrance and exit was chosen. The data (campaign BC-C2) are presented in Fig. 3. *T*_cave_ showed regular peaks (about 0.7 °C high) corresponding to the individual tours (Fig. 3a). The values repeatedly increased from about 9.6 °C, reached a maximum of about 10.4 °C, and finally returned to baseline after the visitors left the chamber. In contrast, only hints of peaks were found on the curve of *T*_DP_ varying in a narrow range between 9.0 and 9.4 °C. In contrast to the BC-C1 campaign, the *T*_ext_ values showed a systematic decrease from 10.7 to 8.9 °C. Similarly to *T*_ext_, the resulting $\Delta {T}^{{\text{virt}}.}$ values showed a systematic decrease from the initial value of 0.9 °C to the minimum value of − 1.3 °C after the last visit tour left the chamber. The presence of individual visitor groups caused decreases in $\Delta {T}^{{\text{virt}}.}$ values up to 1.8 °C. The evolution of $\Delta {T}^{{\text{virt}}.}$ values indicated the presence of DAF ventilation mode until the arrival of the second visitor group switching the cave ventilation into UAF ventilation mode. *RH* shows an inverse evolution with respect to *T*_cave_ (Fig. 3b): individual tours led to an absolute decrease in *RH* values by 4%. After the visitors left the chamber, the *RH* values returned to the initial value of about 98%. Inversely to *RH*, *AH* shows that the input of visitor tours leads to an increase of up to 0.1 g m^−3^. After conversion from *RH* to *AH*, the data show an increasing trend similar to *T*_cave_/CO_2_: visitor input induces an increase of around 0.1 g m^−3^. After the visitors left the chamber, the *AH* values returned to the initial 9.0 g m^−3^. The inputs of individual visitor tours corresponded to CO_2_ concentration increases: individual net increments, Δ ${c}_{{{\text{CO}}}_{2}}$, varied between 263 ppmv (the first tour) and 161 ppmv (the last tour) (see Fig. 3c). Between the entries, CO_2_ concentrations decreased but did not return to the previous values leading to the total increase of 510 ppmv (from 820 to 1330 ppmv).

#### Monitoring under the regime with high number of visitors

##### Monitoring campaign VC-C1

**Fig. 4:**
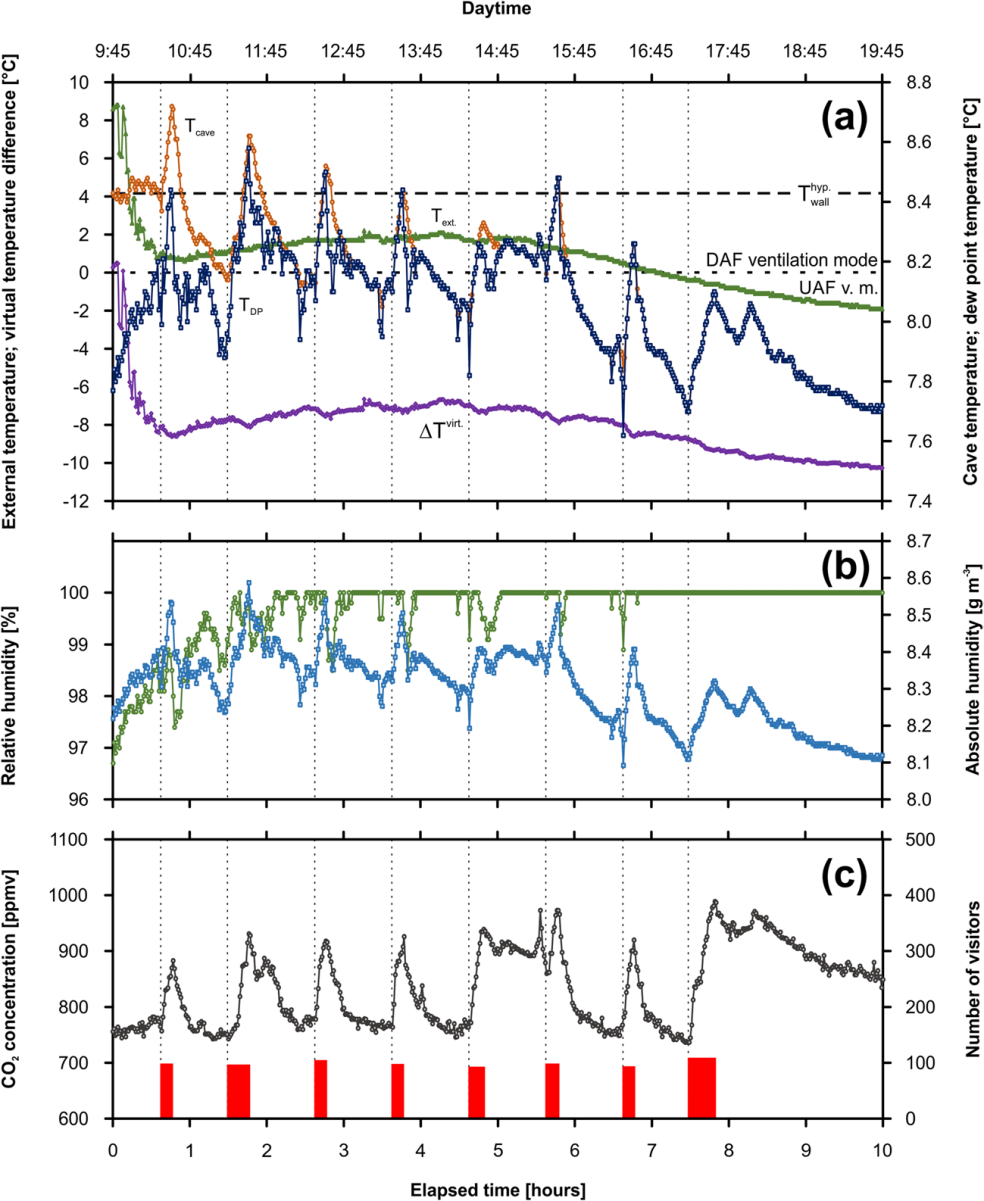
Monitoring campaign VC-C1 (30 November 2019; Kotelna Chamber, Výpustek Cave): the evolution of cave air/dew-point/external temperature and virtual temperature difference (**a**), cave air relative/absolute humidity (**b**), and CO_2_ concentration (**c**) in dependence on the individual tours of visitors (the widths of columns represent the time visitors spent in the chamber)

For this purpose, Výpustek Cave (MK) was chosen in order to use cultural events with an average attendance of around 100 people per tour. Monitoring data from a Kotelna Chamber showed sharp peaks corresponding to the entries of 8 individual tour visits of 794 people in total (Fig. 4). The *T*_cave_/*T*_DP_ showed almost the same trends, however with different initial values: whereas the values of *T*_DP_ fluctuated about ~ 7.8 °C, the *T*_cave_ showed enhanced values of about 8.4 °C (Fig. 4a). On the other hand, both variables showed anthropogenic peaks between 0.3 and 0.5 °C, consistent with the entries of the visitor group. A linear decrease trend is visible in the temperature evolution. The evolution of *T*_ext_ values showed three periods with different slopes: (i) the initial period (~ 0.7 h) with a rapid decrease of values from 8.8 to 0.8 °C, (ii) the middle period (~ 4.5 h) with relatively constant values about 1.0 °C, and (iii) the systematical decrease of values up to − 1.9 °C. A similar trend was observed in the case of $\Delta {T}^{{\text{virt}}.}$. An initial period was associated with the sharp decrease from 0.3 up to − 9 °C supporting the cave air circulation in UAF ventilation mode. On the other hand, relatively constant $\Delta {T}^{{\text{virt}}.}$ values varying in the range of ⁓ 1.5 °C were measured in the remaining monitored period. The *RH* values gradually increased from 96.7 to 100% during the initial 1.5 h of the campaign. In the remaining period, the *RH* values were 100% except for the presence of visitors, where the *RH* values decreased up to ~ 98.5% (Fig. 4b). As in the previous campaigns, the increase in the *AH* values can be traced depending on the number of visitors. At the highest number of visitors, the initial value of *AH*, 8.2 g m^−3^, increased to the value of 8.6 g m^−3^. A slight decreasing trend is similar to temperature. CO_2_ concentrations varied between 737 and 989 ppmv. The net increase in CO_2_ ranged from 113 to 252 ppmv depending on the number of visitors in each group (Fig. 4c).

##### Monitoring campaign VC-C2

**Fig. 5:**
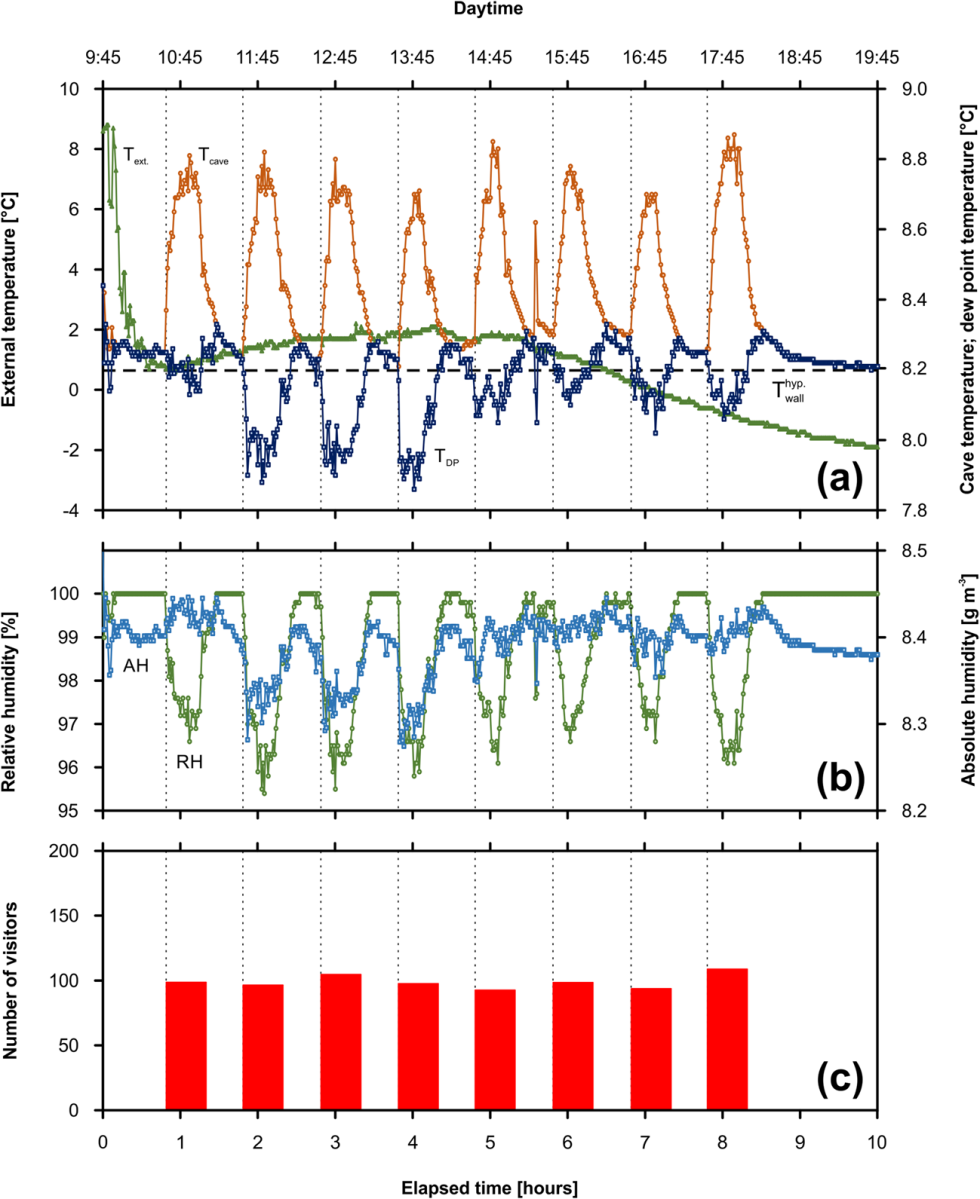
Results of the monitoring campaign VC-C2 at high relative humidity (30 November 2019; Jindřich Chamber, Výpustek Cave): cave air/dew-point/external temperature (**a**), cave air relative/absolute humidity (**b**), and the number of visitors entering the chamber (the widths of columns represent the time visitors spent in the chamber) (**c**)

This campaign in Jindřich Chamber (VC, MK) runs under special conditions with oscillating relative humidity and again high attendance. The evolution of the monitored variables during this campaign (Fig. 5) was similar to the evolution during the previous campaigns, except for the *T*_DP_, where the opposite trend was observed (Fig. 5a). All the variables were strongly influenced by the presence of 794 people divided into 8 visit tours. In the period without visitors, the *T*_cave_ remained around 8.3 °C. With the presence of visitors, the temperature increased by 0.5 °C depending on the number of visitors in individual tours. However, in the case of the *T*_DP_, sharp decreases up to 7.9 °C were identified in the periods with the presence of the visitor. The evolution of *T*_ext_ corresponded to the evolution during the previous campaign. *RH* values in the period without visitors persisted at a level of 100%. In the presence of visitors, these *RH* values decreased to 95.4%. After recalculation, no increase in *AH* was recognized during a visitor presence in contrast to other campaigns: either a constant value or even a decrease was recorded (Fig. 5b).

### The impact on limestone tablets

**Fig. 6:**
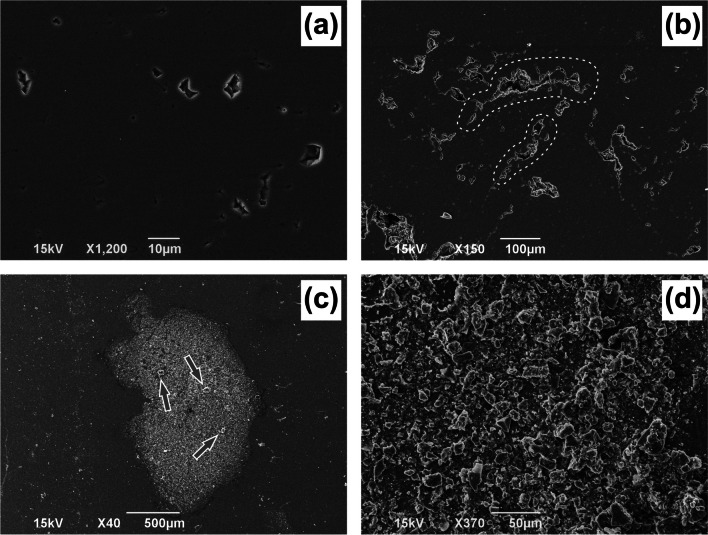
Secondary corrosion of the limestone tablets placed in the Amatérská Cave (Moravian Karst) for the period of 30 months. Total area with natural (**a**) and corroded pores (**b**), traces of calcite recrystallization—gray arrows (**c**, **d**)

The consequence of the corrosion of the limestone tablets by condensed water was studied by the SEM method. The results are given in Fig. 6. The total views showed a natural surface (Fig. 6a) and a finely ragged surface with enlarged pores (Fig. 6b). In the remaining figures, traces of calcite recrystallization in the dry drop (Fig. 6c) and overlaid by cave aerosol (Fig. 6d) are evident.

## Data analysis

### Correlation analysis

Relations between attendance in individual monitoring campaigns and monitored microclimatic parameters (CO_2_ concentrations, air temperature/humidity) were tested using Spearman’s correlation analysis. The BC-C2 modeling campaign was not included in the analysis because of the constant attendance in all visiting groups. Absolute humidity was not used as it depends on the temperature. Therefore, only the relative humidity values were used for the correlation analysis. The resulting correlation between attendance and CO_2_ concentrations was strong (ρ = 0.83) for the BC-C1 campaign, but insignificant (ρ = 0.07) for the VC-C1 campaign. A similar pattern was found in the relationship between attendance and air temperature: there is a strong correlation (ρ = 0.83) for the BC-C1 campaign, whereas the correlations for the VC-C1 and VC-C2 campaigns were insignificant (ρ =  − 0.23). In contrast, a relatively wide range of negative values of the correlation coefficients between attendance and relative humidity were found. The correlation evolved from strong (ρ =  − 0.71 for the BC-C1 campaign) to moderately strong (ρ =  − 0.41 and − 0.50 for the VC-C1 and VC-C2 campaigns, respectively). All correlations are summarized in Table 5.

**Table 5:** Spearman correlations between attendance and microclimatic parameters from individual monitoring campaigns in the Balcarka Cave (BC) and the Výpustek Cave (VC)

Relation	Campaign BC-C2	Campaign VC-C1	Campaign VC-C2
*A* vs. CO_2_	**0.83**	0.07	–
*A* vs. *T*	**0.83**	– 0.23	– 0.23
*A* vs. *RH*	– 0.71	– 0.41	– 0.50

The highlighted correlations are significant at α = 0.05

*A* attendance, *CO*_*2*_ cave CO_2_ concentration, *T* cave air temperature, *RH* cave relative humidity

### Modeling of dynamics of CO_2_ and absolute humidity

**Fig. 7:**
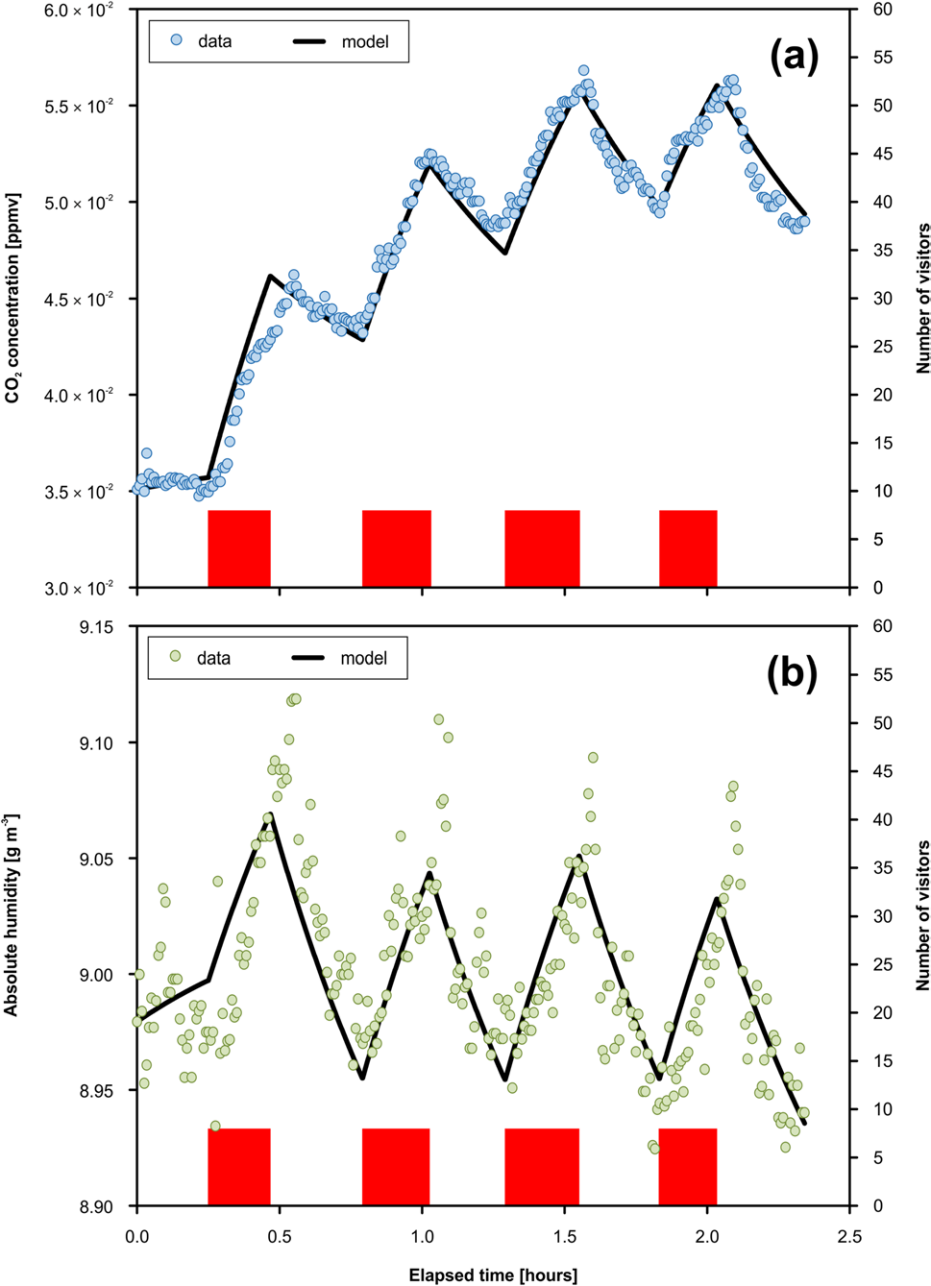
Modeled evolution of CO_2_ levels (**a**) and absolute humidity (**b**) based on the data from the reference campaign BC-C2 (20 November 2019; Museum Chamber, Balcarka Cave)

The modeling of the CO_2_ concentrations resulting from the BC-C2 reference campaign yielded (1) the volumetric velocity through the chamber (airflow) approximately 5.0 × 10^−2^ m^3^ s^−1^ and (2) the total volume of the chamber ~ 126.6 m^3^ (Table 6). The CO_2_ concentration in the chamber adjacent to the monitored chamber, ${c}_{{{\text{CO}}}_{2}}^{{\text{adj}}}$, corresponded to 3.7 × 10^−2^ mol m^−3^. The calculated anthropogenic personal CO_2_ flux, ${j}_{{{\text{CO}}}_{2}}^{{\text{AP}}2}$, was 2.4 × 10^−4^ mol s^−1^. Based on absolute humidity modeling, the water vapor concentration in the adjacent chamber, ${c}_{{\text{WV}}}^{{\text{adj}}}$, reached 9.0 g m^−3^. The anthropogenic personal flux of water vapor, ${j}_{{\text{WV}}}^{{\text{AP}}2}$, was found to be 3.2 × 10^−3^ g person^−1^ s^−1^, and the flux of water vapor associated with condensation, ${j}_{C}$, was determined to be 1.4 × 10^−2^ g s^−1^. The fitting of the data from the BC-C2 campaign (CO_2_ concentrations, water vapor concentrations) by the model curves is presented in Fig. 7.

**Table 6:** Calculated values of cave CO_2_ and absolute humidity (Museum Chamber, Balcarka Cave)

Parameter	Unit	Campaign BC-C1	Campaign BC-C2
CO_2_ modeling			
* v*	(m^3^ s^*−*1^)	5.0 × 10^*−*2^	5.0 × 10^*−*2^
* V*	(m^3^)	126.6	126.6
${c}_{{CO}_{2}}^{adj}$	(mol m^*−*3^)	2.3 × 10^*−*2^	3.7 × 10^*−*2^
${j}_{{CO}_{2}}^{AP2}$	(mol person^*−*1^ s^*−*1^)	2.4 × 10^*−*4^	2.4 × 10^*−*4^
Absolute humidity modeling			
* v*	(m^3^ s^*−*1^)	5.0 × 10^*−*2^	5.0 × 10^*−*2^
* V*	(m^3^)	126.6	126.6
${c}_{WV}^{adj}$	(g m^*−*3^)	9.1	9.0
${j}_{WV}^{AP2}$	(g person^*−*1^ s^*−*1^)	8.9 × 10^*−*3^	3.2 × 10^*−*3^
${j}_{C}$	(g s^*−*1^)	1.5 × 10^*−*2^	1.4 × 10^*−*2^

**Fig. 8:**
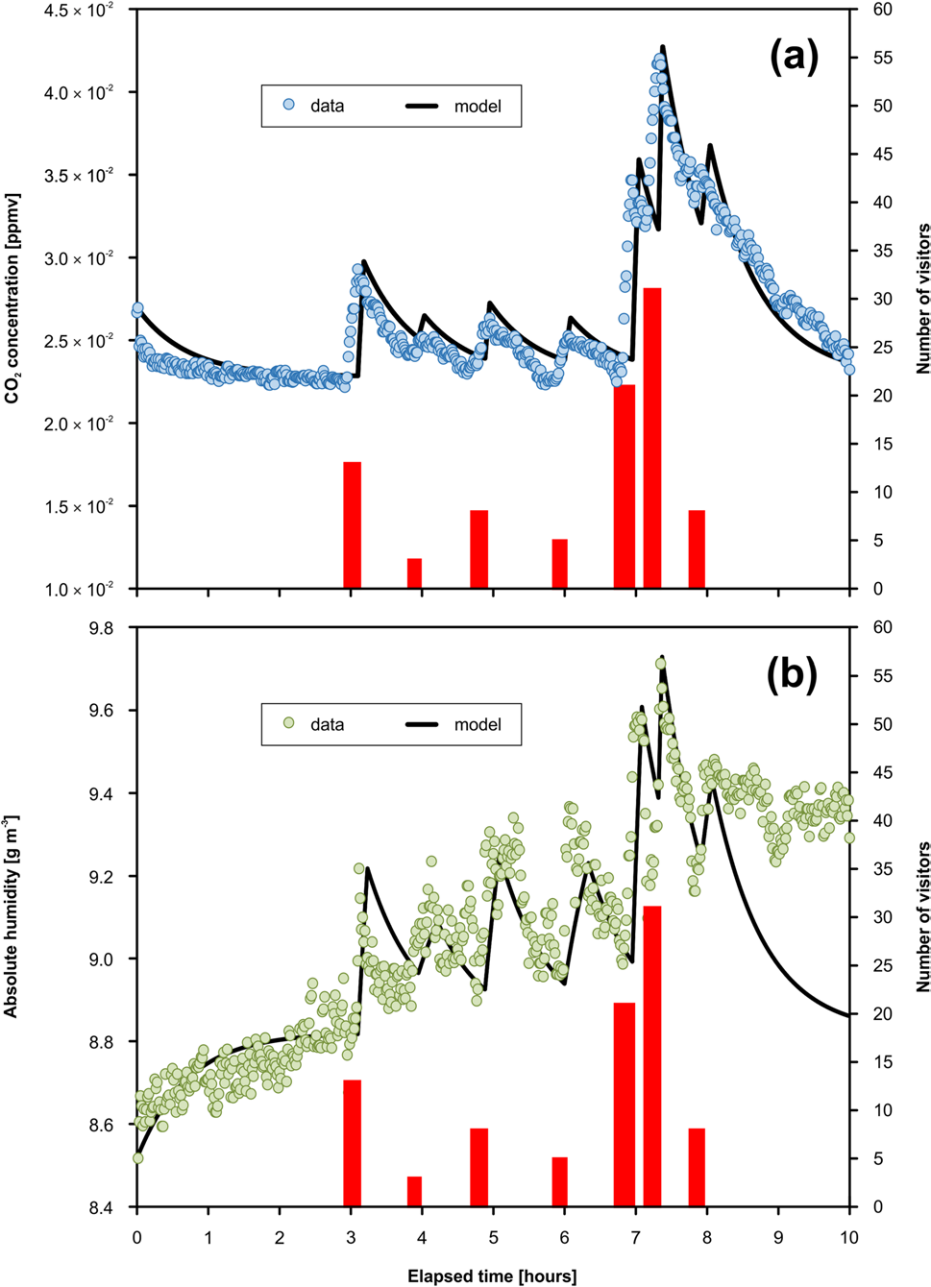
Modeled evolution of CO_2_ levels (**a**) and absolute humidity (**b**) based on the data from the monitoring campaign BC-C1 (22 October 2022; Museum Chamber, Balcarka Cave)

Whereas the BC-C1 monitoring campaign carried out under the standard visit regime showed a lower value of CO_2_ concentration in the adjacent chamber, ${c}_{{{\text{CO}}}_{2}}^{{\text{adj}}}$, of about 2.3 × 10^−2^ mol m^−3^, compared to the reference campaign, it showed a similar value of anthropogenic personal CO_2_ flux, ${j}_{{{\text{CO}}}_{2}}^{{\text{AP}}2}$, corresponding to 2.4 × 10^−4^ mol s^−1^ (Table 6). The model of absolute humidity showed the water vapor concentration in the adjacent chamber, ${c}_{{\text{WV}}}^{{\text{adj}}}$, of 9.1 g m^−3^ and the anthropogenic personal flux of water vapor, ${j}_{{\text{WV}}}^{{\text{AP}}2}$, corresponding to 8.9 × 10^−3^ g person^−1^ s^−1^, and the water vapor flux associated with the condensation, ${j}_{C}$, reached 1.5 × 10^−2^ g s^−1^. The fitting of the data from the campaign BC-C1 (CO_2_ concentrations, water vapor concentrations) by the model curves is presented in Fig. 8.

## Discussion

### Natural conditions

The data representing the natural conditions were monitored during periods without visitor presence (the periods before entry of the first visit tour on a given day) and showed variable CO_2_ concentrations and absolute humidity in the air of different cave chambers. Relatively low CO_2_ levels, between 515 and 858 ppmv, were found in the Museum Chamber and the Kotelna Chamber (Figs. 2c, 3c, and 4c). All these values correspond to the expected levels of CO_2_ measured in BC (Lang et al. 2015a, b; Lang et al. 2017a) and VC (Lang et al. 2017b) and are also roughly consistent with values from other European caves (see, e.g., Baldini et al. 2006; Liñán et al. 2008; Lario and Soler 2010). In addition to the effect of molecular diffusion and water degassing, natural CO_2_ levels in the chambers are controlled by advective CO_2_ fluxes (Lang et al. 2017c). These fluxes are a function of (1) CO_2_ concentrations in the soils in the overburden and external or/and adjacent chamber atmosphere, ${c}_{{{\text{CO}}}_{2}}^{{\text{adj}}}$, together with (2) cave airflows controlled mainly by buoyancy via virtual temperature difference, $\Delta {T}^{{\text{virt}}.}$ (Sánchez-Cañete et al. 2013).

The modeling of data from selected campaigns (BC-C1, BC-C2) in the Museum Chamber (BC) showed a relatively high volumetric airflow rate of 5.0 × 10^−2^ m^3^ s^−1^ (Table 6) typical for the winter season (Bourges et al. 2001; Spötl et al. 2005; Kowalczk and Froelich 2010). However, the almost completely negative values of the virtual temperature difference,$\Delta {T}^{{\text{virt}}.}$, between 0.9 and − 5.2 °C (Fig. 3a) indicate that the Museum Chamber persisted in the UAF ventilation mode. Based on the chamber position, the corridor that passes through the chamber leading from the main exit to the deepest cave passages with an elevation of ~ 11 m was identified as the primary airflow path. External air enters the chamber through the window in the chamber ceiling (partially through the gaps/leakages around the door edges) and flows into deeper cave passages. The Discovers’ Chimney near the main exit could represent a crossroad, where the air flux flows in two directions: (i) through the chimney into the external atmosphere (it could represent a hidden upper cave entrance) or (ii) further toward the deepest cave passages.

The calculated *AH* values showed different values for the individual monitored caves. While relatively higher values between 8.5 and 9.7 g m^−3^ were found in the BC campaigns (Figs. 2b and 3b), lower values of 8.1 to 8.6 g m^−3^ were obtained in the VC campaigns (Figs. 4b and 5b). Similar to CO_2_, the increase in *AH* values in the atmosphere of BC could be associated with advective fluxes from adjacent spaces, controlled by (1) *AH* values in the adjacent cave spaces, ${c}_{{\text{WV}}}^{{\text{adj}}}$, and (2) cave airflows. Based on calculated cave airflows of 5.0 × 10^−2^ m^3^ s^−1^, the ${c}_{{\text{WV}}}^{{\text{adj}}}$ values corresponded to 9.1 g m^−3^ (BC-C1 campaign) and 9.0 g m^−3^ (BC-C2 campaign) (Table 6). Because these values significantly exceed the *AH* values in the Museum Chamber, it could be deduced that the water vapor was transported into the chamber from deeper cave passages. Lower *AH* values monitored in the VC could result from increased relative humidity. This mechanism is evident, especially in the case of the VC-C2 campaign, when the chamber atmosphere is saturated with water vapor (*RH* = 100%) before the entry of the first visitor group (Fig. 5b), and any excess water vapor transported from the adjacent cave spaces could condense on the walls of the cave. If the chamber atmosphere was unsaturated by water vapor, the measured AH values could reach values similar to those found in the BC.

### Anthropogenic impact on microclimatic variables

The anthropogenic impact on CO_2_ and humidity levels is visible in the peaks superimposed on the smoother curves of natural CO_2_ levels (Figs. 2c, 3c, and 4c) (Pulido-Bosch et al. 1997; Carrasco et al. 2002; Šebela et al. 2013; Lang et al. 2015a, b; Lang et al. 2017b). Similarly, the immediate increase in cave air temperature is associated with the presence of the visitor (Figs. 2a, 3a, 4a, and 5a). This is in agreement with, e.g., Lario and Soler (2010) or Calaforra et al. (2003). Although an increase in air temperature leads to a decrease in *RH* values, it increases the AH values. So the presence of individual visiting groups leads to peaks on the natural *AH* curve (Figs. 2b, 3b, 4b, and 5b).

In general, the heights of these peaks are given by (i) the number of visitors in individual visit tours and (ii) the time visitors spent in the chamber (Lang et al. 2017b). However, the data are associated with two uncertainties. The first is the time of the entry of visitors into a given chamber. The movement speed of individual groups of visitors differs depending on the number of visitors and the length of a guided commentary. In this case, the bases of temperature peaks were used as indicators of the entry of the visiting group. The second uncertainty results from the time visitors spend in the chamber, as the time is not constant for all groups and might depend on the group’s size. This phenomenon may have manifested especially during the BC-C1 campaign: the Museum Chamber represents the last stop on the guided tour of Balcarka Cave. The visitors can leave the chamber (and cave) on their own, after they have seen the exhibition. Despite these uncertainties, the correlation analysis showed moderate to strong correlations between the number of visitors and the anthropogenic CO_2_ (ρ ~ 0.83), *T* (ρ ~ 0.83), and RH (ρ ~  − 0.71) in the Balcarka Cave (Table 5). However, the mentioned uncertainties could operate in the case of the data from the Výpustek Cave, where all the correlations were found to be insignificant except the relation between the number of visitors and *RH* (ρ ~  − 0.41 to − 0.50) (Table 5). Moreover, it could also be associated with a narrow range of visitor numbers (up to 10 people) between individual visiting groups and/or the position of monitoring chambers outside of the main airflow path (Fig. 1).

A comparison of the CO_2_ and *AH* data from BC-C1 and BC-C2 campaigns with the model curves showed some differences between both variables. While the CO_2_ concentration data fitting by the model curves was relatively close in both campaigns (Figs. 7a and 8a), some inconsistencies were identified in the case of *AH* data during both the natural and anthropogenically influenced monitoring periods. These inconsistencies are visible in the case of (i) the highest values of the anthropogenic peaks, especially during the BC-C2 campaign (Fig. 7b) and (ii) the final period of the BC-C1 campaign (Fig. 8b), where the *AH* values measured in the cave chamber significantly exceed the modeled values. While the modeled lower values of *AH* are associated with the relatively low total volume of 127 m^3^ (Table 6), corresponding to the so-called effective volume (ventilated part of the chamber), the higher measured *AH* values indicate a higher total volume of the chamber (cumulation of the excess of *AH* in the non-ventilated chamber spaces around cave walls, e.g., niches).

The mean personal flux of CO_2_ into the Museum Chamber, ${j}_{{{\text{CO}}}_{2}}^{{\text{AP}}2}$, of 2.4 × 10^−4^ mol person^−1^ s^−1^ (Table 6) is roughly consistent with the values of 2.9 × 10^−4^ mol person^−1^ s^−1^ presented by Faimon et al. (2006) or (2.6–3.4) × 10^−4^ mol person^−1^ s^−1^ reported by Milanolo and Gabrovšek (2009). This ${j}_{{{\text{CO}}}_{2}}^{{\text{AP}}2}$ values fall within the wide range of values from 6.7 × 10^−5^ mol person^−1^ s^−1^ (Lang et al. 2015b) to 1.5 × 10^−3^ mol person^−1^ s^−1^ (Dragovich and Grose 1990) reported from show caves worldwide. Such a wide range of CO_2_ fluxes probably results from differences in human age (Tormo et al. 2001), activity (Iwamoto et al. 1994), and gender (Sciacca et al. 2002). The mean personal flux of water vapor into the Museum Chamber, ${j}_{{\text{WV}}}^{{\text{AP}}2}$, varied in the range of (3.2–8.9) × 10^−3^ g person^−1^ s^−1^ (Table 6). Generally, personal water vapor flux fluctuates in the range of (0.2–8.0) × 10^−2^ g person^−1^ s^−1^ depending mainly on human physical activity (Christian 1994; Zieliński and Przybylski 2012). Therefore, the resulting ${j}_{{\text{WV}}}^{{\text{AP}}2}$ values in the Museum Chamber indicate the slightly increased physical load of visitors, which corresponds to the complex morphology of the Balcarka Cave (Lang et al. 2015b).

The simulation of microclimate conditions by a dynamic model does not have to fully describe the complete process in the cave. In contact with the cave air, breathed water vapor cools quickly, and a part of the vapor can condense directly in the cave air. This water subsequently settles on the surrounding walls and speleothems and participates in the calcite-water interaction. Just this part of the water vapor is unmeasurable during monitoring. This behavior was confirmed by the wider range of ${j}_{{\text{WV}}}^{{\text{AP}}1}$ values of (0.6–1.2) × 10^−2^ g person^−1^ s^−1^ (resulting from the calculation based on visitor numbers) in comparison with the narrower range of ${j}_{{\text{WV}}}^{{\text{AP}}2}$ values of (3.2–8.9) × 10^−3^ g person^−1^ s^−1^ (the modeled values, see Table 6). Therefore, an alternative approach based on the amount of air exhaled and initial water vapor concentration was used for the calculation of the dissolved calcite amounts.

### Condensed anthropogenic water

Calculations of the mass of exhaled water vapor and volumes of condensed water showed relative differences between individual caves (Table 3). In general, the total mass of water vapor exhaled by visitors in individual MK show caves is given by (i) the anthropogenic personal flux of water vapor, ${j}_{{\text{WV}}}^{{\text{AP}}1}$, (ii) total attendance, and (iii) the time a visitor group spends in the given cave. While the ${j}_{{\text{WV}}}^{{\text{AP}}1}$ value was constant, the other parameters changed. In case of total attendance, an important role played the period of cave accessibility to tourists: whereas the shortest accessibility period (12 years) in VC corresponded to total attendance of 249,249 people, the longest accessibility period of 64 years in PC was connected with total attendance of 15,208,888 people (Table 3). It indicates that attendance represents a key parameter that quantifies the mass of anthropogenic water vapor and the volume of condensed anthropogenic water. However, the comparison of BC and S-ŠC, the caves with the same accessibility period (29 years) and mean tour time (60 min), showed a difference in total attendance by more than 200,000 people but relatively consistent masses of anthropogenic water vapor and only slightly increased volumes of anthropogenic water (Table 3).

Different attendance of individual caves also reflects strong seasonality in the production of anthropogenic water vapor and water. Although a significantly higher summer attendance was identified in all caves (Table 3), the relationship between summer and winter attendance in individual caves changed. Whereas the lowest ratio value of 3.3 was found in VC, the strongest values of seasonal attendance ratios of 14.3 and 14.5 were registered in BC and KC. For comparison, the summer attendances in PC and S-ŠC exceeded the winter attendances by factors of 7.3 and 11.2, respectively. The quantification of the anthropogenic contribution to the total volume of water vapor in the cave atmosphere may be distorted when the cave air is saturated by water vapor or *RH* values are close to 100% (typically in the winter season or after the precipitation events during the summer season). In our study, periods with such conditions were identified: 100% *RH* values were found during the VC-C1 and VC-C2 campaigns (Figs. 4b and 5b) carried out in November. After reaching the saturation of the cave air by the water vapor, the additional amount of exhaled water vapor would immediately condense. At the same time, the total volume of water vapor in the cave atmosphere is reduced by cave ventilation to the values corresponding to the steady state, ${c}_{{\text{WV}}}^{{\text{ss}}}$. In contrast, the lower *RH* in the summer season allows the identification of the total volume of anthropogenic water vapor. The total volume of condensed anthropogenic water vapor during the BC-C1 and BC-C2 campaigns could be quantified by the ${j}_{C}$ values. Based on Eq. (12), the anthropogenic contribution of the water vapor was (0.3–2.8) × 10^−1^ g s^−1^ (BC-C1) and 2.5 × 10^−2^ g s^−1^ (BC-C2). Compared with the appropriate ${j}_{C}$ values indicate that under given conditions, 6–57% (BC-C1) and 55% (BC-C2) of the water vapor exhaled by individual visitor tours would condense on the cave walls.

### Implications

The long-term effect of the calcite dissolution in individual caves follows the trend of anthropogenic water vapor. The total amounts of calcite potentially dissolved in the individual MK show caves varied in a wide range. Based on different physical activity of visitors, the values changed from 1280 to 2561 g in VC; 3160 to 12,732 g summary in BC, S-ŠC, and KC; and 29,519 to 59,038 g in PC (Table 6). Similar to the anthropogenic water vapor and the condensed water, a significant seasonality was also identified in the amount of dissolved calcite. Based on the increased summer attendance, the model mass of dissolved calcite was 4.9 to 21.4 times higher in summer than in winter (Table 4). We tried to estimate the amount of dissolved calcite in an individual cave per year. It is not a simple task because each cave was opened to the public in different years. Records of visitor numbers are also available for different periods, so the impact may be quantified only for these periods (see Table 3 and 4). For calculation, the most visited Punkva Caves were taken. The amount of dissolved calcite per year ranges from 0.39 to 0.78 kg, based on the year season and visitor activity.

The impact in the calcite-water-CO_2_ system is controlled by the kinetics in the phase boundaries of calcite-water and water-CO_2(g)_. In the case of the water film with comparable surface areas of phase boundaries, areas as the kinetics are similar. However, the peak concentrations of CO_2_ resulting from the exhalation of visitors during their stay in the cave are controlled by ventilation and quickly return to their original levels (see Figs. 7a and 8a). Thus, the kinetics of calcite dissolution seems to be more important, as the impact of condensed water acts longer in comparison with anthropogenic CO_2_. However, from a long-term view, the number of visitors with the frequency seems to be crucial.

For all available data on attendances/periods in the MK show caves, the mass of dissolved calcite is greater than 88 kg. This quantity is environmentally risky, especially because of the inconspicuousness of the dissolution process progressing to depths of a few micrometers but operating over large areas. It can be deduced that dissolution by condensed water leads to two consequences: (1) condensation corrosion of calcite (see Fig. 6b) or (2) recrystallization of the calcite/limestone surfaces (see Fig. 6c, d). Both processes are controlled by the transport of the dissolution products. In the first case, the drop formed during extensive condensation dissolves calcite and then drips down from the surface, transporting the products of dissolution away. Another drop forms at the same site, and the cycle repeats, leading to condensation corrosion. Note that the transport of calcite by drops dripping down from the wall is slower than the process of calcite dissolution by condensed water. In the second case, the drop is not large enough and remains on the surface until cave conditions change and the drop evaporates. During evaporation, the solution becomes supersaturated and new calcite growth, which leads to the gradual recrystallization of the calcite/limestone surface. Such a process could be a potential risk for the conservation of prehistoric cave paintings such as in the well-known Altamira Cave (Sánchez-Moral et al. 1999; Gázquez et al. 2022), Lascaux Cave (Guerrier et al. 2019), or other caves worldwide, for example, French Points Cave (Lafon-Pham et al. 2022) or Chinese Mogao Caves (Mikayama et al. 2015).

### Cave management

The results of this work clearly showed that anthropogenic water (water vapor exhaled by cave visitors) can condense and cause calcite corrosion, although such an impact appears to be relatively low. In general, a reduction of calcite corrosion would involve limiting the number of cave visitors or manipulating the ventilation to keep the relative humidity below the dew-point. Since manipulating ventilation is risky due to affecting the cave microclimate as a whole (changing variables such as *T*_cave_, *RH*, CO_2_/Rn levels, etc.), it seems to be the only option to reduce the number of visitors. Deciding on this is generally difficult because it means economic losses and reduced cultural enjoyment to achieve a relatively small reduction in impact on the cave environment. Fortunately, a sensitive compromise is possible: comparing the impact of relatively small groups of visitors in Balcarka Cave (Figs. 2 and 3) with the impact of large groups of visitors in Výpustek Cave during cultural actions (Figs. 4 and 5) indicates that cave environment is just threatened by large groups of visitors. The result indicates that the groups of up to 30–35 visitors under common conditions do not cause water condensation. The time gap between the groups of around 1 h is sufficient to relax the conditions and return the microclimatic variables to their original values, even if their gradual increase is noticeable (see Figs. 2 and 3). Increasing the number of visitors in a group or shortening the spacing between groups is risky in proportion as the number of visitors approaches a hundred in a group where water condensation has already been clearly demonstrated (see Figs. 4 and 5). It should be emphasized that cave protection depends to a certain extent on local conditions, such as the geometry of cave spaces, the slope of the floor, the number of entrances, the nature of the soil cover, and climatic conditions. Therefore, it would be ideal to fine-tune the cave management based on its own measurements in the spirit of this work.

When it comes to caves with historical artifacts, such as artistic drawings on the walls, they are threatened not only by direct corrosion but also mainly by the gradual crystallization of secondary calcite (see Fig. 6c, d). The newly grown calcite is in danger of obscuring the paintings, which can lead from reduced visibility to complete overlay and essentially the disappearance of the painting. In this case, the number of visitors to the cave must be regulated particularly carefully.

## Conclusions

The anthropogenic impact of CO_2_ and water vapor on cave speleothems was studied in the show caves of the Moravian Karst (Czech Republic). The main findings resulting from this work are as follows:

- Natural CO_2_ and absolute humidity levels were mainly controlled by advective fluxes associated with cave airflows.
- The presence of visitors led to peaks superimposed on the smoother time curves of natural microclimatic variables. Visitor peaks could be distorted by some uncertainties associated with (i) the time of entry into a given chamber, (ii) the period of their stay in the chamber, and (iii) the position of the monitoring chamber outside of the main ventilation routes of the cave.
- The production of water vapor was simulated using the simplified dynamic model based on the mass fluxes of the relevant variables. Model results could be underrated because of the fact that part of exhaled water vapor might condense directly into cave air, immediately after exhale. For this reason, a simplified approach based on the theoretical amount of exhaled air and the concentration of water vapor in the breath was used to calculate the dissolved calcite.
- The total volume of water vapor exhaled annually by visitors in the Moravian Karst Show Caves was controlled by attendance and tour duration, in addition to microclimatic parameters. The total exhaled water vapor could be increased by a factor of five in dependence on attendance. The tour duration can increase the volume of the anthropogenic water vapor by a factor of two.
- The calculation of the mass of dissolved calcite showed significant seasonality: the mass dissolved in summer exceeded the mass dissolved in winter.
- Condensation to a higher extent participates in the so-called condensation corrosion, which requires the removal of dissolution products.
- The impact of the calcite-water-CO_2_ system is controlled by the kinetics and interaction areas of the phase boundaries of calcite-water and water CO_2_. In the case of the water film, the kinetics and areas are similar. While anthropogenically influenced CO_2_ peaks quickly return to their original levels due to ventilation, the impact of condensed water acts longer.
- Condensation to lower extents without the removal of dissolution products could cause the recrystallization of calcite. The effect could be a potential risk, for example, for prehistoric cave paintings.
- The results indicate the importance of improving the cave management to keep the cave microclimatic parameters close to natural conditions. Possible proposals could be to reduce the number of visitors in groups and/or shorten the period of cave tours.

## Data Availability

Data are available from the corresponding author upon request.
